# Essay upon the Pathological Anatomy of Stricture of the Urethra

**Published:** 1843-07-01

**Authors:** 


### I. MEMOIRE SUR L'ANATOMIE PATHOLOGIQUE DES RETRECISSEMENS DE L'URÉTRE. Par le Dr. *Civiale*. 1842.

Essay upon the Pathological Anatomy of Stricture of the Urethra.

### II. MEMOIRE SUR L'EMPLOI DES CAUSTIQUES DANS QUELQUES MALADIES DE L'URÈTRE. Par le Dr. *Civiale*. 1842.

Essay upon the Employment of Caustic in some Affections of the Urethra.

THESE pamphlets may be regarded as appendices to Dr. Civiale's large work upon the Diseases of the Genito-urinary Organs, and, as we have so recently furnished a copious analysis * of that excellent production, we will content ourselves with a very brief view of the present Essays, and the more readily, because, short as they are, they contain many repetitions of what we have already brought under our readers' notice, in the numbers referred to.

### I. PATHOLOGICAL ANATOMY OF STRICTURE OF THE URETHRA.

Since the publication of his work, Dr. Civiale has visited our metropolis, and subjected the numerous pathological collections it contains to a minute inspection. He has here found ample confirmation and illustration of the doctrines and precepts that he had published, and takes frequent occasion to remark upon the richness of our museums in valuable specimens of every variety of urethral disease. This, however, is not to be looked upon in the light of a compliment, but the reverse, as he believes

---

* See Medico-Chir. Rev. for July and October, 1842.

much of our pathological wealth has arisen from our faulty practice. Thus, speaking of false passage, he thus expresses himself—

"When I visited the rich collections of London, I was struck with the prodigious number of preparations, shewing every variety of false passage. I communicated to one of the first surgeons of that city, who accompanied me, the reflections so frightful a collection gave rise to. 'You need not be surprised,' he replied, 'to find here a greater number of examples of false passage than elsewhere, for it arises solely from the greater care with which we preserve the preparations of the accident: the number of such cases that occur is pretty much the same in all parts.' So far from partaking this opinion of my London *confrere*, I am, on the contrary, persuaded that the principal cause of such multiplicity is the manner in which catheterism is performed, and the hazardous means which are had recourse to in order to force the stricture. In no place have such means so extensively prevailed as in London, and in no place has less attention been paid to the practice of catheterism—an operation often confided to the assistants. To understand how this department of surgery has been neglected by the English it is sufficient to look at the curvatures of their instruments, for with such it must be difficult if not impossible to avoid making false passages."

The author considers that the curve of the instrument should be much slighter and more prolonged.

Protesting altogether against the accuracy of the criticism upon the manner in which catheterism is usually conducted in this country, we are inclined to believe there is more truth in the explanation offered by the London Surgeon than Dr. Civiale is disposed to admit. Judging from the falsification of facts as to the result of cases, and the manner in which post mortem examinations are made to furnish the desired confirmations, on the Continent, we should feel little expectation of finding there the same manly candour, which prevails in our own country, in avowing, and exhibiting for the warning of others, the results of unsuccessful practice. Moreover, when we remember the praiseworthy zeal and indefatigable industry with which the directors of our museums search out for illustrative examples, it is nowise suprising that they should have accumulated a great number. We consider it no reproach to possess such collections, but we regret to record our conviction, that, rich as they are in every variety of anatomical and pathological illustration, got together with great industry, maintained in the most perfect order at great expense, and thrown open to all enquirers with the greatest liberality—they are far too little frequented both by students and practitioners. Our author, by reason of the unrivalled dexterity his long practice and great opportunities have given him, possesses the right of criticism in a full degree, but we can assure him, that, although there may be some truth in his remarks upon the curvature of our catheters, &c., yet, that they are by no means the formidable instruments which he seems to suppose, and that the production of false passage by their employment is of rare occurrence, in the hands of a careful surgeon, (M. Civiale himself acknowledges this is frequently unavoidable even under the most skilful management,) while the practice of forcing strictures, save in some great emergency, never has been admitted as legitimate practice. Of course the urethra has its quacks, licensed and unlicensed, as every other organ of the body has, but our author's work amply shews that such, bearing no mean names, exist also in his own country. To proceed with our brief analysis:

Several circumstances conspire to prevent pathological anatomy throwing all the light that can be desired upon stricture. In many patients, who have fallen victims to the immediate consequences of stricture, the nature of these has never been examined, and it is only quite recently that attention has been directed to them. "The pathological facts, too, collected in many localities, especially England, have been turned to little account. It is not without surprise that I have seen in the London museums great numbers of documents lost as it were to science." Although the greater number of fatal diseases of the bladder are the direct or indirect consequence of stricture, yet, owing to the gravity of these secondary lesions, they absorb all the attention of the practitioner, and the state of the urethra, especially as the patient is frequently enabled still to urine, is overlooked. After death, too, the narrowing of the canal is frequently not found to be proportionate to the difficulty experienced in passing instruments during life. Again, on examining the urethra, one is often surprised to find so inconsiderable a lesion of a surface, which may have abundantly secreted puriform matter for years, and to which repeated applications of caustic have perhaps been made. Lastly, it is not so often in the actually strictured part itself, as at a point posterior, and sometimes anterior to it, that organic changes are to be found: for the narrowed and dense part has remained untouched, while, the urethral pouches between it and the bladder, have had to bear all the impulse produced by the endeavours to evacuate the latter organ, and those anterior to the stricture have been submitted to the application of instruments.

The *Lesions constituting the Stricture* having been so recently described in the article alluded to, we may pass on to the section treating of *Organic Lesions resulting from Stricture.* In estimating these, we must bear in mind that two very different conditions of the bladder may exist in persons afflicted with this malady. Sometimes, its walls are hypertrophied, and possess a great expulsive power, while, in other cases, they are often in a condition of atrophy, when the organ possesses less than its natural power of contracting upon its contents. As the altered condition of the parietes of the urethra, situated posteriorly to the stricture, is chiefly produced by the impulse of the column of urine, it is easy to see that the extent to which this takes place will be much influenced by the condition of the bladder. The principal effects produced are (1), *Phlegmasia* of the mucous membrane of the *urethra* posterior to the stricture. The discharges so often found in stricture proceed from this locality rather than from the strictured portion of the canal itself, or from the tumified condition of the prostate, and it is here too that the morbid sensibility of the canal is especially found. Indeed, the inflammatory condition of the membrane, which preceded the formation of the stricture, seems to recede afterwards to a point posterior to it. Here it may be followed by the usual sequelæ, as softening, ulceration, partial destruction. Sometimes the urethra is pierced like a sieve, giving rise to several urinary fistulæ, but, in most cases, a solitary perforation exists. (2). *Abscesses,* situated in the substance of the urethral parietes, may follow the destruction of its lining membrane. (2). The *dilatation* of the urethra, behind the stricture, varies much in size, and sometimes becomes so large as to be mistaken for the bladder itself. Great dilatation of the mucous follicles, seated near the neck of the bladder, and of the orifices of the spermatic and prostate ducts likewise occur, and that more frequently and extensively than is usually imagined. (4). The partial destruction of the mucous membrane also often leads to the formation of small sacculi in the urethra, especially at the inferior and lateral parietes of its membranous portion. (5). *Ruptures and lacerations,* sometimes occurring spontaneously in the membranous portion, are usually preceded by inflammatory action. (6). Changes not infrequently occur in the portion of the canal *anterior to* the *stricture,* especially near the glans, arising from the sympathy of continuity of structure. (7). *Lesions of the prostate,* whether effects or mere coincidents, are very frequent accompaniments, and exert a powerful effect in increasing the gravity of the malady, and the difficulty of relieving it. We will not follow our author in his detail of the varieties of these, as his observations form but a condensed repetition of what he has stated at large in his former work; but we may remark, judging from the detailed and somewhat elementary character of the demonstrations he lays so much stress upon, as not generally understood by his readers, that the nature of these changes, the obstacles they present, and the means by which they are to be overcome, must be far more familiar to our practitioners than they are to their brethren on the Continent—a result due, no doubt, to the admirable series of preparations illustrative of these points which most of our schools possess. (8). *Lesions of the Bladder.* These vary according as the walls become hypertrophied or the contrary, and whether catarrh may have co-existed for a greater or less time. (9). *Of the Ureters.* These are usually irregularly dilated, and their parietes, though not often thickened, are reddened. (10). *The Kidneys.* Most patients who die in consequence of stricture do so from the kidneys having become implicated.

*False Passage.*—The author observes that, when we consider the extent, situation, and direction of strictures, the deviations the canal behind them undergoes, the form and size of the instruments employed, the various special modes of treatment had recourse to, and the want of experience of many of those who endeavour to treat the affection,—we should rather feel astonished that so few examples of false passage exist than at their abundance.

Athough it may occur in the anterior portion of the urethra, it generally takes place, where the mobile joins the fixed portion, just under the arch of the pubes. Usually it is the inferior wall of the urethra that is perforated, and, when the accident has occurred to the upper, it has generally arisen from lowering the handle of the instrument too soon, so that its point is thrust against the upper portion of the wall of the urethra. But, even when the instrument has passed this point safely, the precise time when, in cases of bad stricture, its point deviates from the passage is by no means so easily distinguished as books would lead us to suppose; for, the supposed sensation imparted to the hand of the surgeon, or felt by the patient, the occurrence of hæmorrhage, or the change of direction the handle of the instrument undergoes, often afford little or no information, and, in spite of every precaution, the most skilful practitioner will sometimes commit the fault of inducing false passage. His difficulty becomes very great when he is called in to examine a case, in which the false passage has perhaps been recently effected by others, for he will now run great risk of becoming saddled with the malpraxis of his predecessor.

### II. Employment of Caustic.

It would seem, from the observations of the author, that the practice of treating stricture by caustic, once so much in vogue and now so generally abandoned in Britain, is still recommended by practitioners of repute in Paris, in spite of the little success which has attended it. He gives a brief historical sketch of the various means which have been employed to bring the caustic substance in contact with the stricture, many of which quite fail in this object, and others pre-suppose a degree of perviousness of the strictured part, which should render the use of caustic unnecessary. When M. Civiale has had occasion to use this substance, he has usually succeeded by a modification of some of the old procedures, rather than by the adoption of any of these complicated apparatus. He selects a wax bougie of a size suited to the stricture, and rolls an inch of its extremity in powdered nitrate silver, wiping off with a piece of rag any of the caustic which may not have become blended with the wax. This, protected by a canula, is passed down to the stricture, and the requisite extent of the armed point is glided as far within it as a previous examination with bougies has proved to be practicable or desirable. This is the best mode when, as is occasionally useful, it is desired to cauterize an extensive and indurated surface of stricture, which has resisted dilatation, and whose low degree of vitality may call for stimulation. It is also useful in some of the gleety discharges which have their seat in the deep portion of the urethra, or at the neck of the bladder. Each application should continue but a minute or two, and the same bougie will serve for more than once.

Among the advocates for caustic there is a discrepancy of opinion as to the nature of the action or effects which it produces. Most consider it rather as a means of modifying the vital action of the canal preparatory to dilatation, than of directly destroying the obstacle. Others look upon the caustic as operating a true destruction of the stricture, and consider all subsequent dilatation as useless or injurious. The latter restrict its use to linear stricture, and consider that when the affection is extensive it is contra-indicated. Some apply it in the slightest manner, while others continue it for five minutes or more. Some recommend we should not have recourse to it until the situation, extent, direction, and number of the strictures have been carefully ascertained, while others think these preliminaries are useless, and, if there are several strictures, attack them simultaneously if possible. Some, too, unless a favorable result at once follow the application, refrain from repeating it, but, others, let the result be what it will, continue the applications even to *hundreds of times*. Practically we should be guided by the nature of the stricture, in deciding whether caustic is applicable, and the author is of opinion that its use can be extended to a very limited number of cases, while, applied as it is at present, in France, it leads to much injury. In the simple bridle (1) occupying only a portion of the circumference of the urethra, a slight application of caustic is often followed by as slight a re-action, and the affection is forthwith relieved, or a large bougie may at once be passed. (2). Even when the case is more advanced, and the stricture has acquired some consistence, one, two, or even three very slight, short, and sufficiently interrupted applications are sometimes attended with beneficial results. (3). When a stricture is very considerable far less amendment usually follows a first application, and still less and less each subsequent one, and if, in spite of this, the practice be persisted in, the case becomes aggravated and far more serious. In cases (4) wherein the stricture, situated at the anterior part of the canal, is sufficiently considerable to prevent the application of caustic to any of those which may be situated behind it, yet these latter often become benefited to a considerable degree by applying the caustic to merely the anterior stricture, and, therefore, without having themselves come in contact with the substance used. This, among other things, proves that the caustic cannot act by its mere power of destroying the tissues it touches. Its escharotic effect has, indeed, been far too readily assumed, for, if, as has been said, each application is followed by a loss of substance, the one or two hundred cauterizations, performed by some practitioners, should destroy even the parietes of the urethra as well as the mucous membrane, which lines them. As the essential cause of the stricture does not arise from an accidental production, developed upon the surface of the mucous membrane, capable of removal by an escharotic, but from a change in the organization of those parts which the mucous membrane covers, to influence this directly by caustic it would be requisite that such covering should be destroyed. But when the mucous membrane has become partially removed, from any cause, various serious accidents, as infiltration, fistulæ, &c. have resulted. Post-mortem examinations show that the application of caustic leaves no traces upon the surface. The part touched becomes reddened and puffed, and in a few days detaches a greyish pellicle —a modification of the sensibility of the part and an increased capillary circulation occurring for the time. When the caustic produces more violent effects, it has been too freely applied, and is hurtful. In most cases, when the application has been properly conducted, the pain is insignificant, and, indeed, in many cases, either from the nature of the means employed or from the quantity of discharges existing in the canal, it is doubtful whether the substance comes into contact with the stricture at all.

The author is disposed to almost confine the use of caustic to the case of simple bridle, for, if in more serious and extensive stricture, a temporary amendment results, it is usually followed by an aggravation of all the symptoms, sooner or later; and, besides the danger of inducing false passage and undue inflammatory action, its frequent use is followed by a thickening of and loss of elasticity of the walls of the urethra, so that the difficulty of urining continues, although perhaps the urethra may be sufficiently capacious to admit a large instrument. Indurations of a greater or less extent, a gleety discharge of obstinate continuance, severe pains in the region of the neck of the bladder and vesiculæ seminales, and loss of virile power are other consequences too frequently observed. Dr. Civiale severely criticizes M. Lallemand, who formerly a frequent practiser of cauterising, as the grand means of cure of stricture, has not, now he has found reason to modify his practice, made his change of opinion sufficiently known; so that many practitioners still follow, upon his authority, a practice which he himself has much modified. The author also notices, to blame, the practice of indiscriminately applying caustic, whether in solid form or strong solution, to the neck of the bladder in cases of blennorrhagia.

Both the pamphlets are amply illustrated by short details of cases and references to preparations in the London Museums; and, in respect to the use of caustic, the author declares that he is constantly consulted by persons, who, temporarily relieved by other practitioners by this means, after a few months, have found themselves suffering under a new and aggravated train of symptoms, induced by the injury done to the urethra; whose cases have been rendered infinitely worse, and whose complete cure has become hopeless.

